# Obituary

**Published:** 1920-05

**Authors:** 


### OBITUARY

### A TRIBUTE TO THE MOMORY OF DR. JAMES McMANUS

(By the Hartford Dental Society)

With deepest regret we record the death of our professional brother—our staunch and true friend—Dr. Jas. McManus.

He was known the world over as one deeply interested in the progress and elevation of the dental profession and was prominent in establishing the fact that Horace Wells was the discoverer of anaesthesia. He took an active part in our national society and was held in the highest esteem. We believe the records of dental conventions throughout the country for a period of many years would show the name of Dr. James McManus as having taken an active part in all vital matters pertaining to the profession, more frequently than that of any other man in the country. He was an organizer of the Connecticut State Dental Society, and its first president, and his interest in the society never abated. He was a charter member of the Hartford Dental Society, and has given it his valuable counsel and conscientious support during all the years of its existence, and was rarely ever absent from its meetings as long as he was able to attend.

His memory will always be cherished by his many friends, and he will be remembered by the Hartford Dental Society as one of its greatest benefactors.

He will be greatly missed.

Gilbert M. Griswold,
Nelson J. Goodwin,
Charles H. Riggs,
Committee.

